# Bayesian estimation reveals that reproducible models in Systems Biology get more citations

**DOI:** 10.1038/s41598-023-29340-2

**Published:** 2023-02-15

**Authors:** Sebastian Höpfl, Jürgen Pleiss, Nicole E. Radde

**Affiliations:** 1 Institute for Systems Theory and Automatic Control, University of Stuttgart, Pfaffenwaldring 9, 70569 Stuttgart, Germany; 2 Institute of Biochemistry and Technical Biochemistry, University of Stuttgart, Allmandring 31, 70569 Stuttgart, Germany

**Keywords:** Computational biology and bioinformatics, Systems biology

## Abstract

The Systems Biology community has taken numerous actions to develop data and modeling standards towards FAIR data and model handling. Nevertheless, the debate about incentives and rewards for individual researchers to make their results reproducible is ongoing. Here, we pose the specific question of whether reproducible models have a higher impact in terms of citations. Therefore, we statistically analyze 328 published models recently classified by Tiwari et al. based on their reproducibility. For hypothesis testing, we use a flexible Bayesian approach that provides complete distributional information for all quantities of interest and can handle outliers. The results show that in the period from 2013, i.e., 10 years after the introduction of SBML, to 2020, the group of reproducible models is significantly more cited than the non-reproducible group. We show that differences in journal impact factors do not explain this effect and that this effect increases with additional standardization of data and error model integration via PEtab. Overall, our statistical analysis demonstrates the long-term merits of reproducible modeling for the individual researcher in terms of citations. Moreover, it provides evidence for the increased use of reproducible models in the scientific community.

## Introduction

In the last decades, the wide use of computationally intensive methods has shaped all scientific fields. This includes for instance the development of large models, the investigation of these models via simulations, the analysis of big datasets and the integration of data into models. Systems Biology, as an interdisciplinary research field that investigates complex biological processes by combining experiments and mathematical models, has contributed substantially to this development. Novel measurement technologies in the life sciences and an increase in computational resources have triggered the development of large computational models in biology and medicine that are mainly investigated via simulations and multi-step computations. Results of such modeling studies cannot appropriately be reproduced by merely relying on textual descriptions of models and methods in scientific publications, which has led to intensive discussions about standard formats and reproducibility.

Recent systematic studies about the reproducibility of scientific results have uncovered many problems and non-reproducible results, which have coined the term reproducibility crisis^1^. For example, in a nature survey, 70% of the researchers indicated that they failed at least once to reproduce a scientific result of another researcher^2^. Begley et al. stated that there exist several non-reproducible landmark papers in oncology^3^, which build the basis of ongoing research but it has never been tried to falsify their results. These studies raised awareness on the topic in the scientific community. Especially in interdisciplinary life science areas, problems about reproducibility have been reformulated as challenges in the last years, with constructive ideas and measures to address those towards a joint vision of a reproducibility culture^4–7^.

Systems Biology is at the forefront in developing standards for data and model sharing according to the FAIR (Findable, Accessible, Interoperable and Reusable) principles^8,9^. Particularly the reproducibility of models, which is the focus of this study, is fostered by standard model formats that enable the exchange of models between platforms and researchers. Documentation via standardized annotations improves accessibility and interoperability^10^. Furthermore, making these models findable, e.g., via public databases, is key to reusability. Systems Biology contributes by (i) developing standard formats for models such as the Systems Biology Markup Language (SBML)^11,12^ or CellML^13^, (ii) providing formats for simulation descriptions such as the Simulation Experiment Description Language (SED-ML)^14^, (iii) making use of databases such as BioModels^15^, JWS Online^16^ or FAIRDOMHub^17^ for the long-term storage and annotation of models according to minimum information guidelines, (iv) developing tools and formats such as pyPESTO^18^, pyABC^19^ and PEtab^20^ for parameter estimation and data integration, and (v) providing standards for making whole workflows accessible^21^ such as promoted by the COmputational Modeling in BIology NEtwork (COMBINE) initiative^22^ or the FAIRDOM consortium^23^.

**Figure 1:**
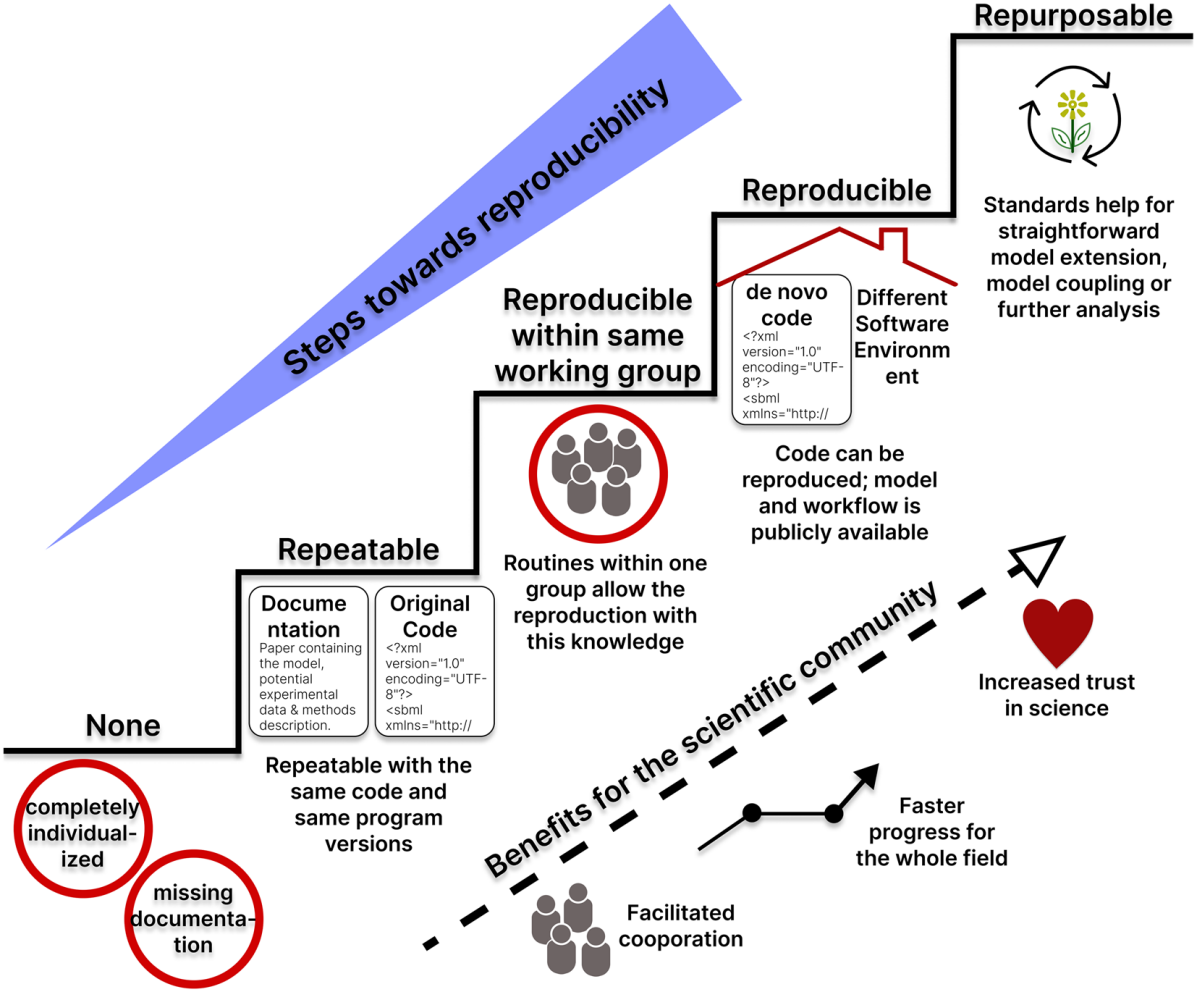
Steps towards reproducibility of computational models in Systems Biology. The lowest staircase represents models and computations which are not reproducible since they are based on completely individualized decisions and implementations with missing or incomplete documentation. Steps towards reproducibility improve the situation towards repeatable models, then models which are reproducible within the same working group, and reproducible models that can be reused by peers in the scientific community. Finally, repurposable models can easily be extended and coupled with other models^6,51^. The benefits of reproducibility for the scientific community are, among others, facilitated cooperation, faster progress in the research field and increased trust in science.

Figure 1 illustrates steps towards the vision of fully reproducible modeling adapted to computational models in Systems Biology and related fields (see e.g., Mikowski et al.^24^). We adopt the definitions for repeatability and reproducibility from Tiwari et al.^25^, according to which repeatability is “the ability to use the same code provided with the manuscript and the same software to reproduce the simulation results” and reproducibility is “the ability to build the code *de novo* and/or ensure the mathematical expressions are correctly represented and reproduce the simulation results in a software different from the one originally used”. Furthermore, the true value of reproducibility is realized when the models are reused or extended for a new purpose. In our viewpoint, the step from reproducible to reusable modeling concerns a commitment to common standards of the whole community, such that models can be easily exchanged and re-used by peers. Individual researchers can contribute by adhering to standards to make their work reproducible. Long-term benefits of this development for the scientific community are productive collaborations and facilitated falsification, which lead to faster progress for the entire field, and ultimately, an increased trust of peers and the public in scientific reasoning.

Although the long-term advantages of reproducible results are obvious, making one’s own results reproducible requires considerable effort from the individual researcher, as well as a sustainable concept for maintaining established workflows and continuous training in the research group. An essential question is therefore that of benefits for the individual researcher which serves as motivation to develop such a concept. The vision of completely repurposable models is to be easily findable and accessible, such that they can broadly be reused in different contexts. According to this vision, we hypothesize that reproducible models are easier to reuse and thus get more attention, resulting in higher citation rates.

This study is based on data from Tiwari et al.^25^, in which the reproducibility of 455 ordinary differential equation models of various biological processes from 152 journals were analyzed. These models were selected in conjunction with the curation process of the BioModels^26^ repository, one of the largest open-source repositories for quantitative models of biological systems. Strikingly, the authors found that only about half of these models were directly reproducible, meaning that at least one main model simulation figure from the respective scientific manuscript could be reproduced by encoding the model in SBML (or checking the SBML file) and using a simulation software different from the original one^25^.

Here, we adopt a fully Bayesian approach for decision-making to detect differences between reproducible (group 1) and non-reproducible models (group 2). In conventional Null Hypothesis Significance Testing (NHST), decisions are based on p-values that control type I errors. A type I error occurs if the null hypothesis is true but rejected, which is typically controlled by the significance level $\alpha$. In contrast, the Bayesian approach treats all quantities of interest as random variables, which are described by probability distributions. These distributions can be inferred from the posterior distributions of the model parameters and contain additional information that is not available in a purely Frequentistic approach, for example, correlations between parameters. Decisions in the Bayesian context are made through summary statistics of posterior distributions, such as the differences of means between two groups. Specifically, here we use the Bayesian Estimation Supersedes the t-Test (BEST) method^27^, which can handle outliers in the data, as well as different sample sizes and different standard deviations of the two groups. Furthermore, in this framework, it is possible to accept the null hypothesis by defining a Region of Practical Equivalence (ROPE) for the effect size, in which the observed differences are judged to be too small to be practically relevant.

## Results

### Bayesian estimation supersedes the t-test (BEST) applied to citation counts of Systems Biology models

**Figure 2:**
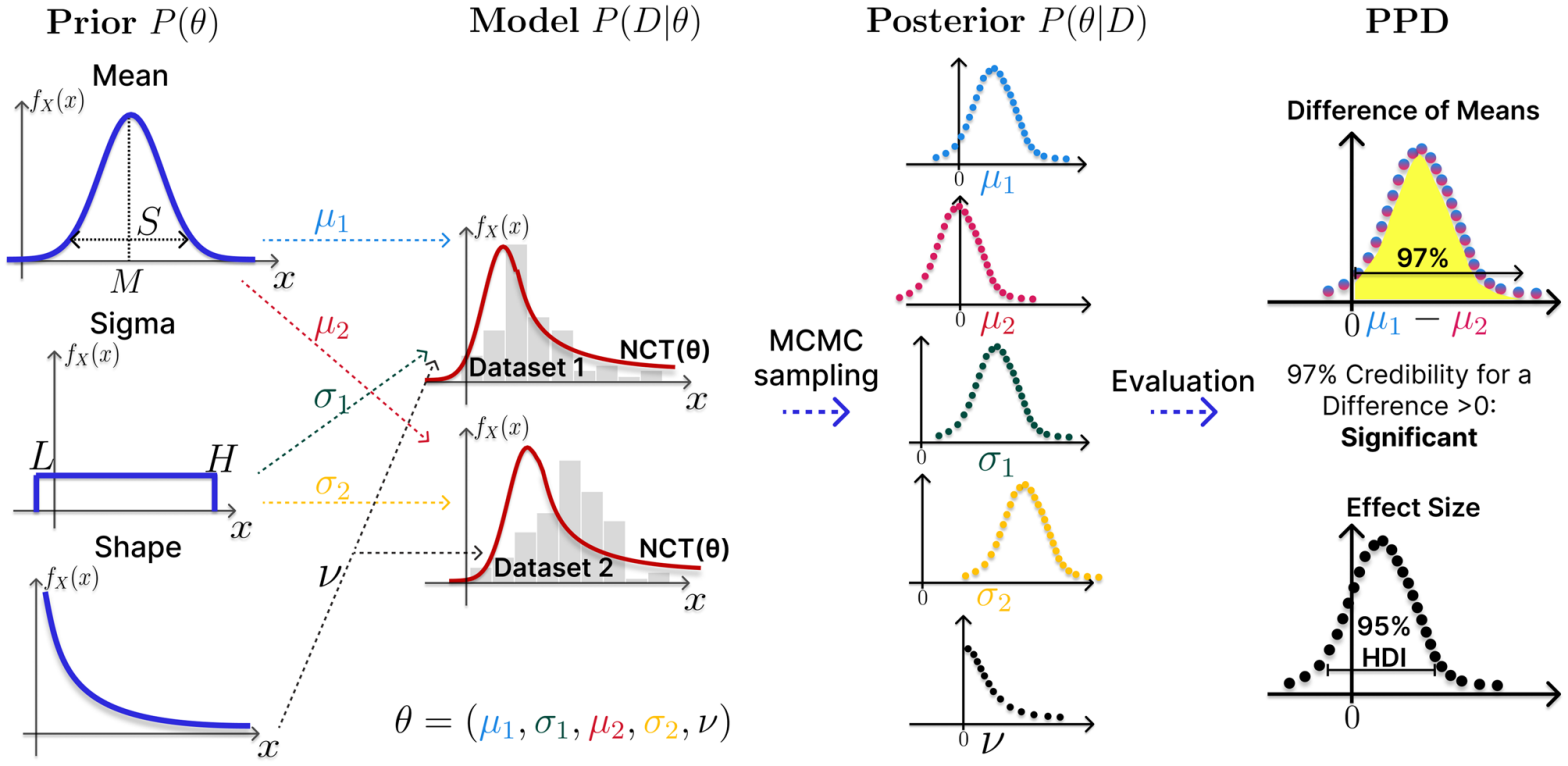
Decision-making in Bayesian estimation is based on credible values. We use the BEST method of Kruschke^27^ for Bayesian estimation. The data $P(D|\theta )$ is described by an NCT distribution. The prior distribution $P(\theta )$ on the parameters is based on the pooled data of both groups. With a normal distributed prior for the mean and a uniform distributed prior for the sigma parameter. The mean *M* of the normal distribution equals the mean of the pooled data and the standard deviation *S* equals 10 times the pooled standard deviation. The lower *L* and upper *H* bound for the uniform distribution equals 1/100, respectively 100 times the standard deviation of the pooled data (see Methods Section). MCMC sampling is used to infer the parameter posterior $P(\theta |D)$, which is illustrated as marginals for individual parameters here. Derived quantities for decision-making are the Posterior Predictive Distributions (PPDs) for the differences in the means of the two groups, $\mu _1-\mu _2$, and the effect size. The null hypothesis of equal means is rejected if 95% of the highest credible values (95% HDI) of the PPD mass for $\mu _1-\mu _2$ is either positive or negative. This means that at least 95% of the values with the highest credibility must lie completely above or below zero to reject the null hypothesis. A Region of Practical Equivalence (ROPE) was defined from $-0.2$ to 0.2 in the effect size, to assess if the difference is of practical relevance. The null hypothesis can be accepted if the 95% HDI lies completely inside the bounds of the ROPE.

In order to test if the citation numbers of papers with reproducible and non-reproducible Systems Biology models differ, we applied Bayesian estimation to the data of Tiwari et al.^25^. The BEST method is illustrated in Fig. 2. Data *D* consist of citation counts of models classified by Tiwari et al.^25^ into reproducible (Dataset 1) and non-reproducible models (Dataset 2). They are described by t-distributions with parameters $(\mu _1,\sigma _1,\nu )$ and $(\mu _2,\sigma _2,\nu )$, which defines the stochastic model $P(D|\theta )$. A statistical test for the choice of this distribution family and the choice of the prior $P(\theta )$ are described in the Methods Section. The independence of all data points is assumed in order to calculate the likelihood value for a particular parameter set. Posterior distributions $p(\theta |D)$ for the parameters $\theta =(\mu _1,\sigma _1,\mu _2,\sigma _2,\nu )$ are investigated via Markov Chain Monte Carlo (MCMC) sampling. Decision-making is based on properties of the Posterior Predictive Distributions (PPDs) of the difference $\mu _1-\mu _2$ and of the effect sizes. The effect size normalizes this difference to a difference in standard deviations (e.g., a mean effect size of 0.5 implies that there is a difference of 0.5 standard deviations between the two groups on average). The null hypothesis that data of both groups have the same mean is rejected to a significance level $\alpha$, if the ($1-\alpha$)% Highest Density Interval (HDI) of the posterior probability mass for $\mu _1-\mu _2$ lies completely above or below zero. This is even more restrictive than taking just ($1-\alpha$)% of the credibility mass of the posterior difference of mean above zero, as the decision is made on the values with the highest credibility only. In the end, this means that often more than ($1-\alpha$)% total credibility for $\mu _1$ being larger than $\mu _2$ must be given to make a decision. Still, precise credibility of different means can be given, which shows better interpretability of the Bayesian method compared to Frequentist hypothesis testing, as a precise number for the credibility can be provided in addition to making a decision based on the 95% HDI. Furthermore, if $(1-\alpha )$% of the credibility mass of the effect size is inside the Region of Practical Equivalence (ROPE), the null hypothesis can be accepted. Such a decision is not possible in Frequentistic approaches. Here, the ROPE analysis was only used as a decision criterion to accept the null hypothesis as it makes the decision criterion too restrictive for small and medium-sized datasets. That is, with the decision criterion of the 95% HDI it is already required in many cases that there is more than 95% total credibility for a different mean. The application of a ROPE further narrows this criterion by requiring the 95% HDI not only to exclude zero but to also exclude the limits of the ROPE. The Bayesian Analysis Reporting Guidelines (BARG) were applied^28^ to make our analysis reproducible and transparent.

### Reproducible models are significantly more cited 10 years after the introduction of SBML

For our analysis, data was processed by assigning a citation count and a journal impact factor (JIF) to each publication from the set of models analyzed in Tiwari et al.^25^ (see Methods Section). The entire processed dataset (1985–2020) consists of $n_R=186$ papers with reproducible (R) and $n_{NR}=142$ papers with non-reproducible (NR) models and spans a period of 35 years (1985–2020). During this period, tremendous development has taken place in the way how researchers search the literature for scientific results and in the availability of computational power and resources. In 1985, standard formats and open-source repositories for model deposition and sharing were not yet a focus in the scientific community. On average, the size of the models was much smaller than today, and many models could still be assessed by carefully reading the corresponding publication. For example, the five top cited papers in this study, with on average more than 600 citations each, are all published before 2008 and were classified to be reproducible^29–33^. The models of these papers are all small, with up to 5 species except the one of Lee et al.^32^ with 16 species. Furthermore, their citation count was probably also promoted by the hot topics they account for, which include HIV, cancer biology and malaria. However, the situation has tremendously changed over time. Today, due to computational resources and experimental data available for model calibration, the majority of modeling publications include larger and more complex models. In order to reproduce, disseminate and re-use these models, textual information is often no longer sufficient and also not a convenient way of handling. Moreover, the programming environment became more complex as different programming languages and toolboxes got popular over time, with many conflicting versions.

To account for this development and to base statistics on a more coherent dataset, we decided to also analyze citation counts of papers that have been published in 2013 or later, i.e., beginning 10 years after the introduction of SBML^11,12^, which has been accepted as the de facto standard format in the Systems Biology community. The publication of SBML is thereby representative of the time when reproducibility in Systems Biology received considerable attention and the first broad standardized solutions for reproducible modeling in Systems Biology were developed. Also, the release of the CellML specifications falls roughly into the same period^34^. This filtering resulted in a subset of $n_{R13}=111$ papers with reproducible (R13) and $n_{NR13}=76$ papers with non-reproducible models (NR13), published since 2013 to 2020.

**Figure 3:**
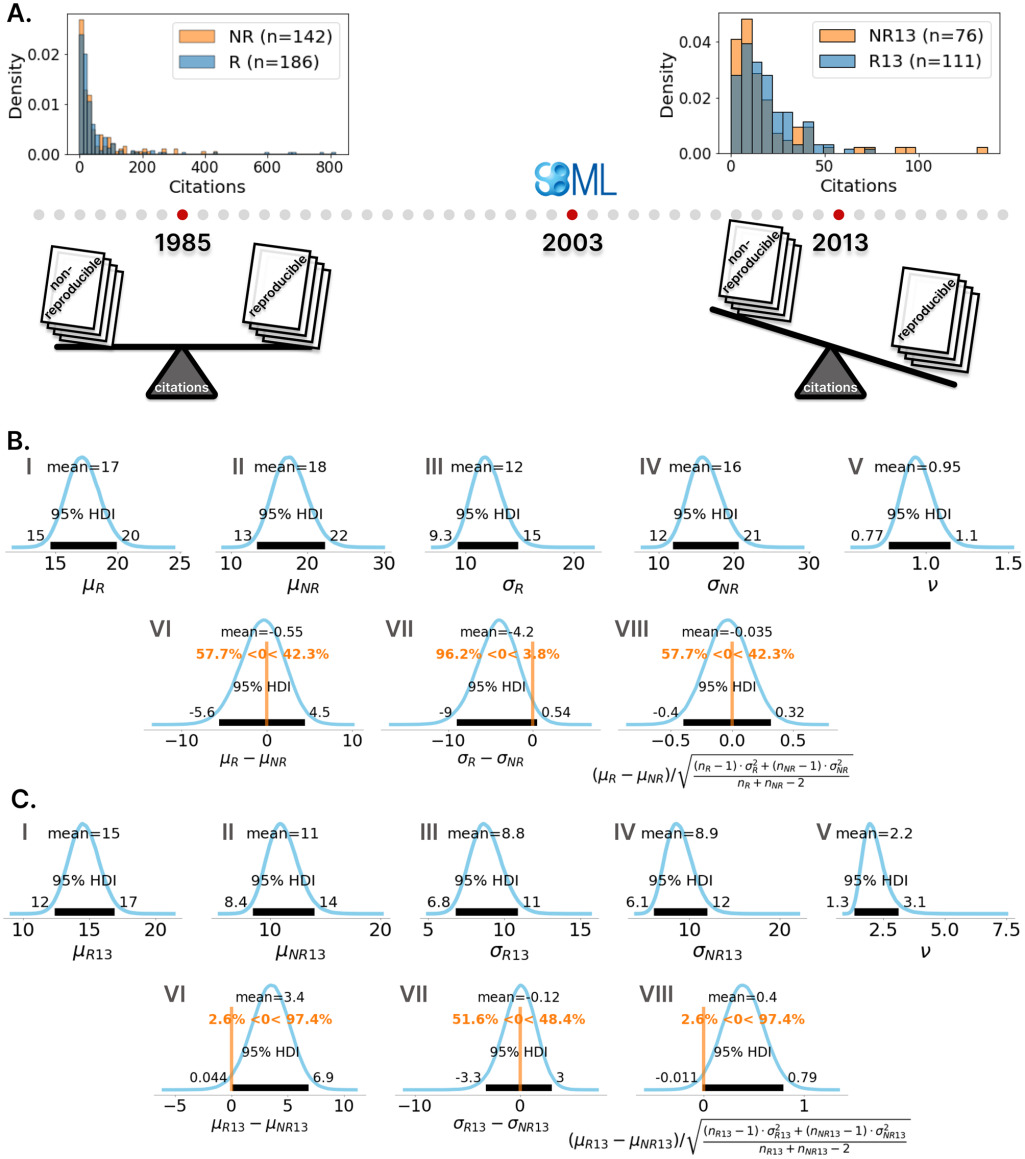
Ten years after the introduction of SBML, reproducible papers are significantly more often cited than the non-reproducible ones. (**A**) Histograms of citation counts normalized to a total area of 1 for each group. The 1985–2020 dataset comprises $n_R=186$ reproducible and $n_{NR}=142$ non-reproducible papers. The 2013–2020 dataset comprises $n_{R13}=111$ papers with reproducible and $n_{NR13}=76$ papers with non-reproducible models, published since 2013. (**B**) Posterior and posterior predictive distributions (PPD) of the period 1985–2020. (**C**) Posterior and PPD of the period 2013–2020. (**B**) & (**C**). Posterior distributions of the reproducible (I) and non-reproducible (II) means $\mu$, reproducible (III) and non-reproducible (IV) standard deviations $\sigma$ and shape parameter $\nu$ (V) and the PPDs for the differences of the means (VI), standard deviations (VII) and the effect size (VIII). The respective distribution means, 95% HDI and probability masses above and below zero are indicated for the PPDs. The choice of prior parameters and details of the MCMC sampling and density estimation are described in the Methods Section. The Potential Scale Reduction Factor (PSRFs) and the Effective Sample Size (ESS) for every inferred parameter or chain is listed in Supplementary Table 1.

Histograms of the 1985 to 2020 dataset (R, NR) and the 2013 to 2020 subset (R13, NR13) are shown in Fig. 3A. We posed the question of whether the average number of citations of the set of publications with reproducible models differs from that of the non-reproducible ones. This was tested using the BEST method as described. The results of the Bayesian analysis comprise the posterior distributions for (I, II) the group means, (III, IV) the group standard deviations, (V) the shape parameter of the non-central t (NCT) distributions, (VI) the difference of the means of the distributions of citations for the two groups, (VII) the difference of their standard deviations and (VIII) the effect size (Fig. 3B,C). Considering the complete period from 1985 to 2020, there is no significant difference between citations of reproducible and non-reproducible papers (Figure 3B). The posterior means of the reproducible and non-reproducible papers are in a similar range ($\mu _R=17$ with an 95% HDI from 15 to 20 citations for reproducible and $\mu _{NR}=18$ with an 95% HDI from 13 to 22 for non-reproducible papers). However, the null hypothesis of equal citations for both groups cannot be accepted, as the 95% HDI of the effect size is not within the ROPE. This confirms the assumption of a heterogeneous group for this period, in which reproducibility only gradually gained in importance.

In contrast, in the 2013 to 2020 dataset (R13, NR13) the reproducible models got on average 3.4 citations more than the non-reproducible ones, as indicated by the mean of the distribution of the difference of means (Fig. 3C). This difference is significant, with a 95% HDI that lies completely above zero and 97.4% of the credibility mass indicating a positive difference of means ($\mu _{R13}-\mu _{NR13}$). Moreover, the distribution of the difference in standard deviations suggests that there is no difference in the standard deviations of the distributions of the two groups. The mean of the difference of standard deviations distribution and its 95% HDI are all closely centered around zero. This indicates an effect for reproducible papers in general and not just a boost for exceptional papers. The mean of the distribution of the effect size is 0.4, indicating a relatively large effect according to Cignac et al.^35^. This means that in the 2013–2020 dataset, about 30% more citations could be achieved through reproducible work alone.

**Figure 4:**
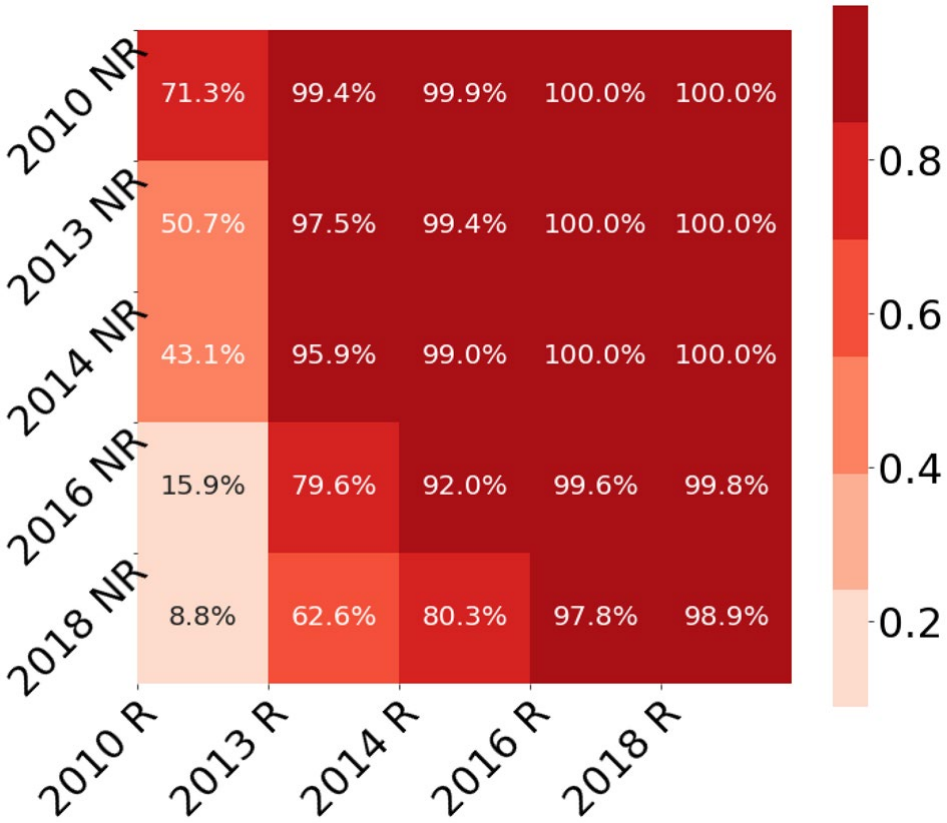
Since 2013, the credibility for higher citation rates of papers with reproducible models remains above 95%. Multi-period comparison for reproducible (R) and non-reproducible (NR) models of credibility towards higher citation counts for reproducible models. The periods 2010–2020, 2013–2020, 2014–2020, 2016–2020 and 2018–2020 were analyzed. Each pairwise comparison shows the credibility for a difference of means above zero for the two groups. The credibilities for a higher mean in the group of the reproducible models are shown.

The choice of the period 2013–2020 was validated by a multiple-period comparison of the periods 2010–2020, 2013–2020, 2014–2020, 2016–2020 and 2018–2020 (Fig. 4). This analysis indicates a tilting point between 2010 and 2013, from a tendency of more citations for reproducible papers with about 70% credibility to clearly higher citation rates for reproducible papers with more than 95% credibility. In all investigated periods starting from 2013, the credibility for a higher citation rate of papers with reproducible models compared to papers with non-reproducible models was between 97.5 and 99.6%.

Overall, the BEST analysis shows that 10 years post SBML, the set of reproducible models is significantly more cited, with an average increase of 3.4 citations.

### The difference in citation rates cannot be explained by differences in JIFs

**Figure 5:**
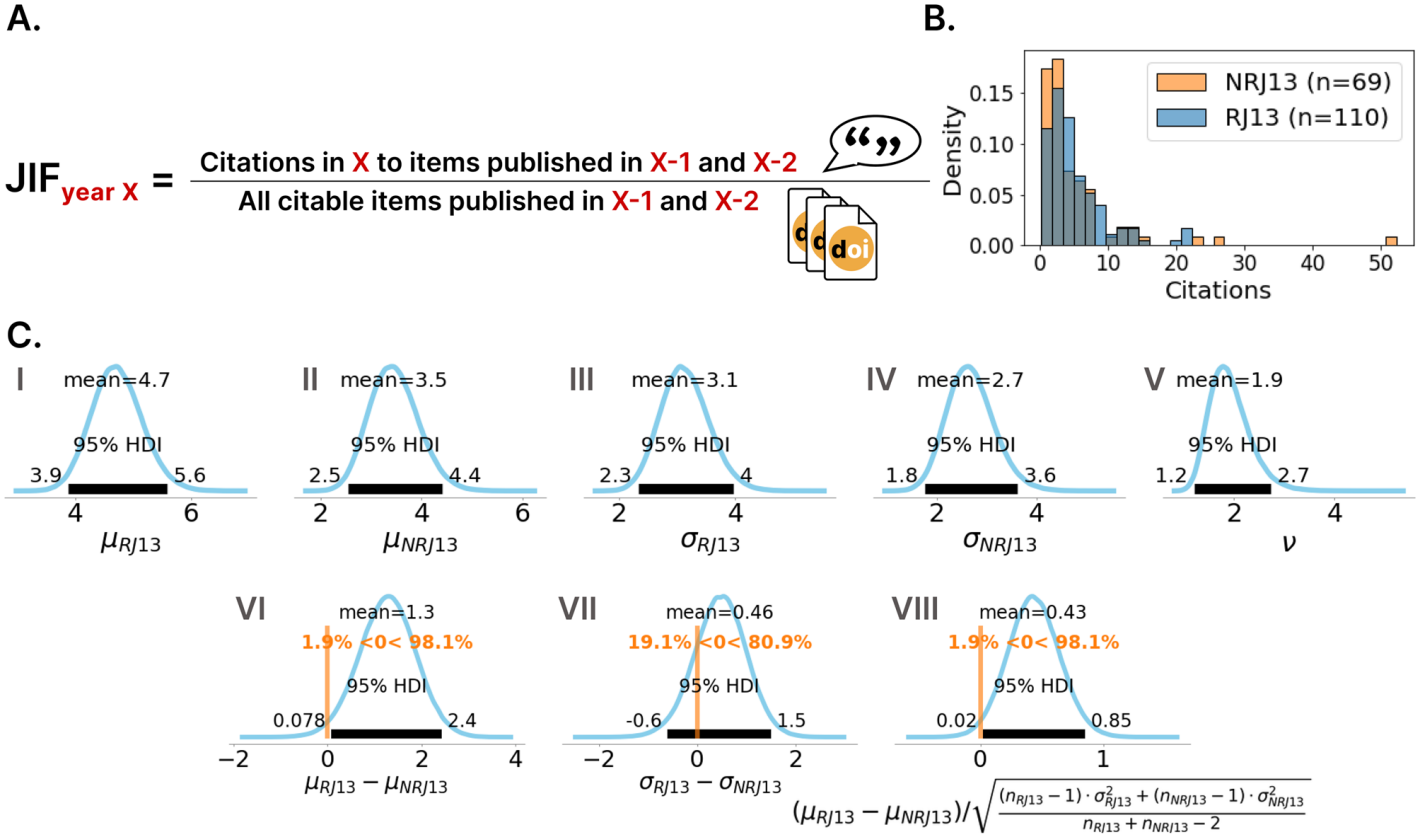
Differences in citation rates among reproducible and non-reproducible models cannot be explained by differences in JIFs. Results of the Bayesian analysis for the 2013–2020 dataset (Fig. 3) were normalized by the respective JIF. A. The JIF of year *X* is calculated by dividing the count of citations, papers published in the two years before ($X-1$, $X-2$) got in these years by the number of all citable items published in this journal in these years. Here, an average JIF of the years 2014 to 2021 of the InCites Journal Citation Report Science Edition of Clarivate Analytics was used. B. Histograms of the reproducible (RJ13) and non-reproducible (NRJ13) JIF normalized citations between 2013 and 2020. The JIF was available for $n_{RJ13}=110$ reproducible and $n_{NRJ13}=69$ non-reproducible papers. The histogram was normalized to a total bar area of 1 for both groups independently. C. Results of the Bayesian analysis (BEST) analogous to Fig. 3.

Next, we asked the question of whether higher citation rates of publications with reproducible models can be explained because these models are predominantly published in journals with higher JIF, which might adhere to higher standards for reproducibility. The JIF is a measure of the average number of citations that a paper in this journal produces one or two years after publication (see equation in Fig. 5A). The JIF was used to normalize the citations to their peer group and to cancel the effects of different JIFs on the number of citations out. Therefore, the citation count of each paper in the study was divided by the corresponding JIF of the journal in which the paper was published (Fig. 5B). As the JIF changes every year, an average JIF of the years 2014–2021 was used to normalize the citations. According to the BEST analysis, the mean of the normalized difference in citations is 1.3, which is significant according to the 95% HDI and the probability mass of 98.1% of difference values larger than zero (Fig. 5C). Again, the two groups do not differ significantly in their standard deviations, and the effect size has a mean of 0.43, which indicates an even stronger effect than without JIF normalization (Fig. 3). Therefore, normalization by the JIF further reinforces the significant trend of more citations of reproducible modeling papers compared to non-reproducible ones. These results show that the difference in citations that were detected in the 2013–2020 data cannot be attributed to the fact that reproducible models might have been published in higher impact journals than non-reproducible ones. This strengthens the assumption that reproducibility itself generates added value in the form of more citations. A significantly higher citation value of the JIF normalized data also indicates an incentive for journals to set stricter rules for reproducibility, as this could increase their impact factor. This shows that the benefit of reproducibility for individual researchers goes hand in hand with the benefit of journals. Summarizing, the observed difference in citation rates between reproducible and non-reproducible models in the 2013–2020 dataset (R13, NR13) cannot be explained by differences in JIFs, since it persists after normalizing the citation counts to the JIFs of the respective journals.

### Additional standardization in data integration correlates with a higher citation count

**Figure 6:**
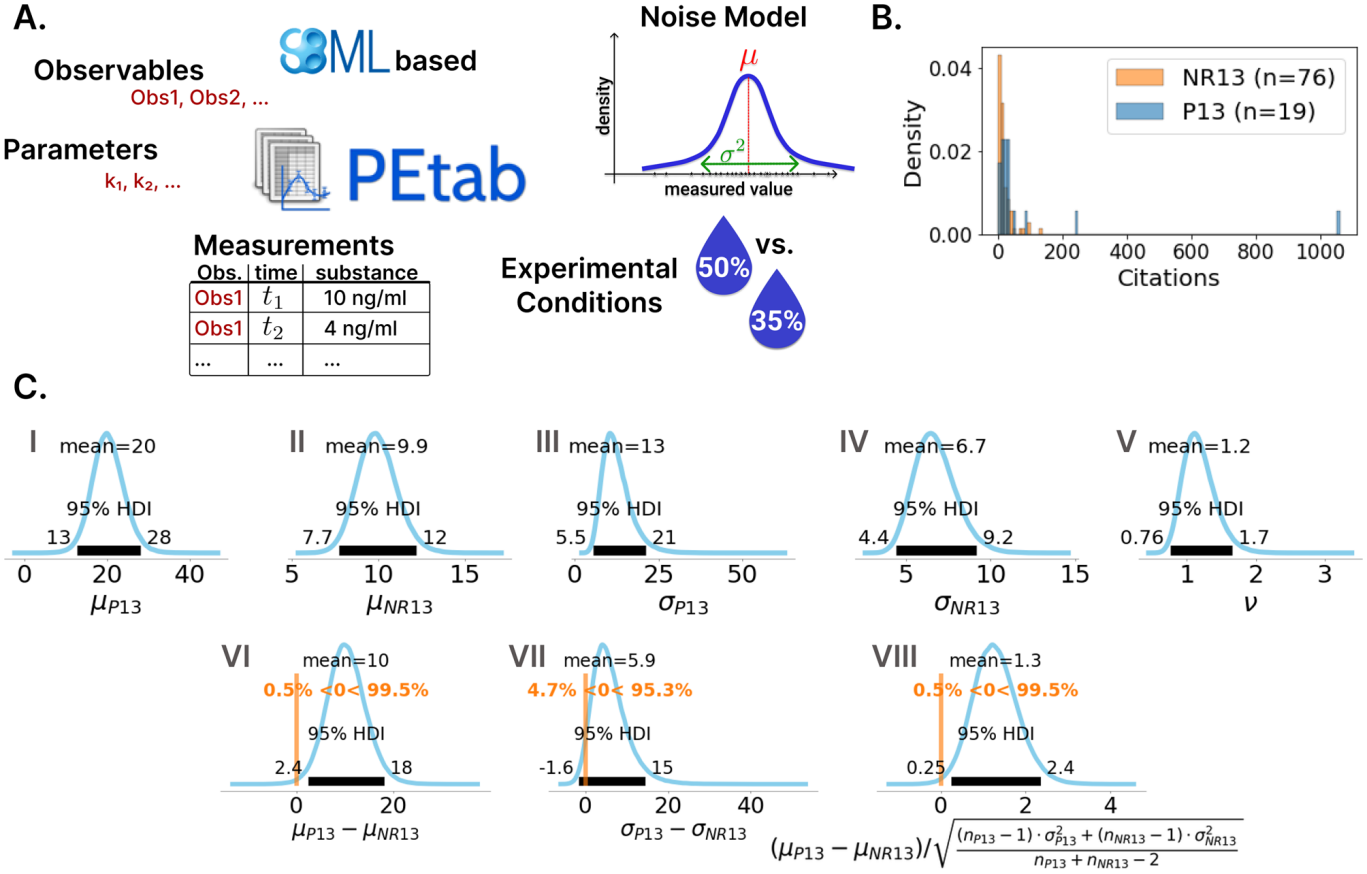
Standards beyond SBML, like PEtab, could further improve reproducibility and repurposability. (**A**) Histograms of citation counts of the PEtab benchmark collection (P13) and the non-reproducible group (NR13) between 2013 and 2020. The histogram was normalized to a total bar area of 1 for both groups independently. (**B**) The PEtab format is SBML based and includes all information to reproduce a complete parameter estimation problem. It consists of five mandatory files, the SBML model, the observables, the experimental conditions, the parameters and the measurement file^20^. Furthermore, a noise model can be specified for maximum likelihood estimation and Bayesian analysis. C. Results of the Bayesian analysis (BEST) to compare the 2013–2020 non-reproducible publications (NR13) and the ones of the PEtab benchmark dataset (P13) analogous to Fig. 3.

Since the advent of SBML, standardization of the modeling workflow has been further promoted. In particular, regarding the process of data integration and parameter estimation. This trend shows a common effort from the Systems Biology community to facilitate the step from reproducibility to reusability (Fig. 1). An example is PEtab^20^ (Fig. 6A), a format that builds on SBML and contains the complete information to reproduce a parameter estimation problem. This includes the computational model in SBML, the experimental data, as well as files for the parameters, observables and experimental conditions and a definition of the noise model. The format can directly be re-used in many python, julia and R tools.

We raised the question whether such models with reproducible calibration procedures correlate with a further increase in citation rates compared to non-reproducible models. For this, we used the PEtab benchmark database (access on 09th May 2022), which includes curated and *de novo* written parameter estimation problems (Histogram in Fig. 6B)^36^. Results of the BEST analysis are shown in Fig. 6C. The papers with completely reproducible parameter estimation problems got on average 10 citations more than the non-reproducible papers. The difference was significant with the 95% HDI and 99.5% credibility indicating a difference above zero. As the posterior mean of the number of citations for non-reproducible papers was 10, a reproducible parameter estimation doubled the average number of citations, with a large average effect size of 1.3.

Interestingly, also the standard deviation was on average 6 citations larger in the PEtab group, with 95.3% credibility indicating a difference above zero. The difference in the standard deviation can be explained in part by the two highly cited papers, which have a large impact on a small sample size of 19. Thus, the trend should be re-examined when PEtab is more established as a standard and a larger benchmark collection exists.

Our result could be a hint that standardization and thereby reproducibility gain importance over time for the visibility of model-based scientific results. However, we are aware that the PEtab benchmark database only contains a few models at the moment and was created after 2013, which is why the effect cannot be attributed solely to the use of the PEtab standard. Moreover, the models in the PEtab Benchmark model database have been manually selected by the toolbox owners and the list contains some popular models, which might also cause a bias in the results.

### NHST Mann-Whitney-U test shows drawbacks of the Frequentist approach

Frequentist NHST tests are the current standard in the biological research field, therefore we asked whether a Frequentist NHST analysis provides comparable results to our Bayesian significance testing. For this, data were first tested for normality via a Shapiro-Wilk test^37^ with a significance level of 5% (Supplementary Figure 1). Since this distribution assumption was rejected, we applied a Mann-Whitney-U test. This is a non-parametric test, at the cost that it only takes qualitative information, i.e., ranks, of the data into account. Results of this testing procedure are shown in Table 1. According to this analysis, for an $\alpha =5$% significance, there is only a difference between the non-reproducible models of the 2018–2020 dataset of Tiwari et al. and the models in the PEtab benchmark dataset. All other combinations do not show a significant difference in their citation counts.

**Table 1:** Results of the Frequentists NHST analysis with the Mann-Whitney-U test. Details of the test are in the Methods Section.

Groups	${n_1}$	${n_2}$	*U* value	*p*-value
1985–2020 Reproducible versus Non-Reproducible	186	142	12920.5	0.6316
2013–2020 Reproducible versus Non-Reproducible	111	76	4718.0	0.0847
2013–2020 JIF normalized Reproducible versus Non-Reproducible	110	69	4296.5	0.0688
2013–2020 PEtab models versus Non-Reproducible	19	76	999.5	0.005
2014–2020 Reproducible versus Non-Reproducible	101	68	3942.0	0.0518
2018–2020 Reproducible versus Non-Reproducible	58	32	1135.5	0.0405

**Table 2:** Summary of the Difference of Means Posterior Predictive Distributions (PPDs) of the BEST analysis.

Groups	${n_1}$	${n_2}$	Mean	95% HDI
1985–2020 Reproducible versus Non-Reproducible	186	142	$-0.55$	$(-5.6, 4.5)$
2013–2020 Reproducible versus Non-Reproducible	111	76	3.4	(0.044, 6.9)
2013–2020 JIF normalized Reproducible versus Non-Reproducible	110	69	1.3	(0.078, 2.4)
2013–2020 PEtab models versus Non-Reproducible	19	76	10	(2.4, 18)

Overall, these results show that there is a discrepancy between the results of the Bayesian (Table 2) and the Frequentist analysis (Table 1). Both methods handle outliers, the BEST method via using NCT distributions and the Mann-Whitney-U test via using ranks. However, the BEST approach provides more information in terms of posterior distributions for all quantities of interest and is thus in our opinion more interpretable. Further advantages of the Bayesian approach are that it is possible to explicitly accept the null hypothesis, parameter correlations can be handled in case of non-identifiable parameters and multi-group comparisons can be conducted without the necessity of alpha correction as false alarms are handled via the model structure in Bayesian hypothesis testing^38^.

## Discussion

In this paper, we have adopted a Bayesian approach for decision making^27^ (Fig. 2) to investigate differences in citation numbers for modeling studies in Systems Biology that were recently classified into reproducible and non-reproducible ones^25^. The raw dataset consisted of 455 models in total and classification was done in conjunction with the curation process of the BioModels repository, i.e., by testing whether at least one figure of the respective manuscript could be reproduced using software different from the one used by the authors. We selected only the models which were directly reproducible (R) and the models which could not be reproduced at all (NR) and assigned citation numbers to them. This resulted in the 1985–2020 dataset with 328 models in total. Tiwari et al. also classified models into the categories “Reproduced with empirical correction” and “Reproduced with author support”. However, these groups were neglected for this analysis as they fall between the categories of clearly reproducible and non-reproducible models. In case of doubt, they require additional effort to be reproduced, which is not always affordable. The 1985–2020 dataset was further processed by selecting a period from 2013–2020 and by assigning JIFs to each publication, resulting in a dataset of 111 reproducible (RJ13) and 76 non-reproducible normalized publications (NRJ13). Bayesian estimation was then applied to analyze differences in citation numbers between the group of reproducible and non-reproducible publications. A difference between the two groups for the 1985–2020 dataset that consists of publications in the period from 1985 to 2020 was not found. However, reproducible models are significantly more often cited between 2013 and 2020, i.e., starting 10 years after the introduction of SBML as a standard modeling format in Systems Biology (Fig. 3). This trend does not depend on the particular choice of the year 2013 and also persists for later periods (Fig. 4).

Our findings are in accordance with other studies, which also emphasize the importance of reproducibility for long-term scientific progress (see e.g.,^24,39^). Possible reasons why reproducible work leads to more citations might be, among others, facilitated cooperation, increased trust in the result and the possibility to reuse the models for other purposes. The effort required to make one’s own results reproducible is often lower than expected. Tiwari et al. provided an 8-point reproducibility scorecard with questions to assist the author in making his/her model reproducible^25^. The authors are of the opinion that the reproducibility of models is already substantially increased even if only half of the questions can be answered positively. The latest version of the scorecard can be found at zendo.

The difference in citation counts even seems to increase with further standardization regarding data integration (Fig. 6). This was observed by comparing the set of reproducible models with a set of benchmark models in PEtab format^20^. This format standardizes also the parameter estimation procedure with experimental data. For publications in this benchmark set the posterior mean was 20 citations, compared to 10 citations for the reproducible ones. As the PEtab database consisted of only 19 papers published in the period from 2013 to 2020 on the 09th May 2022, these results should be validated at a later time point with a larger sample size and publications that use the PEtab format as part of their publication. The use of such standards expands the range of users, as different operating systems and programming languages are supported.

The comparison of the Bayesian with the Frequentist hypothesis testing showed that decisions might differ. As both approaches are valid it is hard to judge which method made the right decision. However, the Bayesian estimation includes more information about the data as outliers are handled via an NCT distribution while the Frequentists Mann-Whitney-U test only includes a qualitative comparison, i.e., ranks. Furthermore, the posterior predictive checks showed that the Bayesian model with an NCT distribution can capture the observed data well (Supplementary Figure 2). In our view, the Bayesian approach through this more comprehensive input information and the richer output information, with explicit posterior distribution of all parameters, is more trustworthy.

Bayesian approaches are powerful, but computationally expensive since the posterior often has to be investigated via sampling, which together with the lack of easy-to-use software tools often prohibits its broad utility^40^. However, increased computational power, combined with the availability of parallelizable toolboxes in C++ like PyMC3^41^, already reduced these prohibitive computational costs, making these methods applicable also to medium size problems. We anticipate that Bayesian approaches will also become available for addressing larger problems in the near future.

## Methods

### Data curation

Data curation was performed in python using the pandas^42^ and numpy^43^ libraries. First, the reproducible and non-reproducible models of the study of Tiwari et al.^25^ were each collected in a separate table. Conference proceedings, preprints and not released models (to 09th May 2022) were excluded as it makes no sense to assign citations to them. Furthermore, if several models of only one published paper were listed, the paper was only counted once for this study. A third table containing the models of the PEtab benchmark collection was added. The number of citations was averaged from Scopus, Web of Science and Google Scholar. Scopus and Web of Science had on average over the complete dataset a 37.13% lower citation count than Google Scholar. Thus, if a paper was not included in Scopus or Web of Science, the missing citation count was replaced by the one of Google scholar times 62.87%. This prevents the betterment of papers that are not listed in the more restrictive databases.

The JIF was taken from the InCites Journal Citation Reports Science Edition of Clarivate Analytics and was assigned to the corresponding papers of the study. Papers with journals that have been closed before 2016 or missing coverage by the Web of Science have been excluded. A list of the papers that had to be excluded can be found on FairdomHub (https://doi.org/10.15490/FAIRDOMHUB.1.STUDY.1103.1). As the JIF is calculated every year, an average JIF of the Journal Citation Reports from 2014 to 2021 was calculated and used for the analysis. The results do not differ qualitatively if only the JIF of 2021 was used.

### Bayesian estimation

For Bayesian estimation, the BEST method of Kruschke^27^ was implemented in python via the PyMC3^41^ library. The method infers posterior mean, sigma and shape parameter values for the investigated datasets by MCMC sampling. To account for outliers, NCT distributions with parameters $(\mu _1, \sigma _1, \nu )$ and $(\mu _2, \sigma _2,\nu )$ were used to describe the data of the two groups. Quantile-Quantile plots (QQ-plots) were used in order to assess whether this is a suitable distribution assumption (Supplementary Figure 3). For this, NCT distribution parameters were fitted to the data via least squares regression with scipy^44^ stats.problot, followed by a two-sided Kolmogorov-Smirnov test^45^ to test the goodness of the fit. The prior distribution for the means $\mu _1$ and $\mu _2$ for the groups of reproducible and non-reproducible models were set to a normal distribution with the sample mean of the pooled data as mean and 10 times the empirical standard deviation of the pooled data as standard deviation. This choice of prior parameters ensures that the probability mass of the prior covers the same order of magnitude as the data and that the prior has a large variance and is thus uninformative and has only little impact on the resulting posterior distribution. In addition, the preference of one hypothesis by the prior is prevented by using the same prior for both groups. The prior of both standard deviations $\sigma _1$ and $\sigma _2$ was set to a uniform distribution with lower and upper bounds of 0.01 and 100 times the empirical standard deviation of the pooled dataset. A sensitivity analysis was performed to validate the results with a broader normal prior for $\mu$ and a broader uniform prior for $\sigma$ with 1000 times the empirical standard deviation of the pooled data as standard deviation, respectively upper and lower bounds (results on FairdomHub). Finally, the prior for the shape parameter $\nu$, which is a measure of the heavy-tailedness of the data distributions and is shared by both groups, was set to a predefined exponential distribution. For the evaluation of the likelihood function $L(\mu , \sigma , \nu )$, we assume independence of all data points, such that the likelihood can be factorized into a product of individual probabilities. The formal specification of the likelihood can be found in the BEST method code on FairdomHub.

The effect size is calculated via Cohens d, with the correction for unequal sample sizes and variances

1 $$\begin{aligned} \sigma _{pooled} = \sqrt{\frac{(n_1-1)\cdot \sigma _1^2 + (n_2-1)\cdot \sigma _2^2}{n_1+n_2-2}}. \end{aligned}$$

A Region of Practical Equivalence (ROPE) was defined in the interval from $-0.2$ to 0.2 for small effect sizes according to Sawilowsky^46^. The null hypothesis can be accepted if 95% of the credibility mass is within this interval. For our purposes, this means that the null hypothesis can be accepted if the limits of the HDI are less than or equal to the limits of the ROPE interval.

A Bayesian multiple comparison method, following the BEST method of Kruschke^27^ was implemented according to the specifications above. Here, the shape parameter $\nu$ was shared between all groups and an individual mean $\mu$ and sigma $\sigma$ parameter was inferred for each group. The difference of means, the difference of standard deviations and effect size was calculated for each pairwise comparison. A heatmap with the credibility values for a group means difference above zero in percent was created to get a good overview of the analysis. The detailed results and diagnostics of the multiple comparison were uploaded to FairdomHub.

To ensure that the Bayesian approach is reproducible and transparent, we applied the Bayesian Analysis Reporting Guidelines (BARG) to the analysis^28^. This includes the calculation of a convergence and resolution statistic via the Potential Scale Reduction Factor (PSRF) and the Effective Sample Size (ESS) with arviz^47^. Furthermore, prior and posterior predictive checks were applied to every Bayesian analysis, to show that the model can capture the observed data (Supplementary Figures 2 and 4, respectively). Choosing a less informative prior, here realized by widening the borders of a uniform distribution, had no influence on the results and shows that the results of the analysis are not sensitive to the chosen prior (results are on FairdomHub). The prior predictive check further shows that the observed data can be captured and the outcome is not biased by the chosen prior.

For MCMC sampling, we used the PyMC3^41^ package version 4.2.2 to create 100,000 samples with 4 chains. A conda environment yaml file, containing all necessary packages to reproduce this study, was uploaded to FairdomHub.

### Visualization

The matplotlib^48^ and seaborn^49^ plotting libraries were used for all statistical visualizations. To visualize the posterior samples of the Bayesian analysis with a HDI probability of 95%, the python package ArviZ^47^ was used.

### Frequentists hypothesis testing

All Frequentist statistical tests were performed in python using scipy^44^ modules. Again, we first tested for normality of the data via QQ-plots, and additionally by performing a Shapiro Wilk test^37^. As groups were not normally distributed according to this analysis, the non-parametric Mann-Whitney U rank test^50^ was applied. The Mann-Whitney U statistic with independent and identically distributed (i.i.d.) samples $X_i$ and $Y_i$ from two datasets *X* and *Y* is defined as

2 $$\begin{aligned} U = \sum _{i=1}^m\sum _{j=1}^n S(X_i, Y_k), \qquad \text { with } S(X_i,Y_i) = {\left\{ \begin{array}{ll}0 &{}\quad \text {when } X_i>Y_i,\\ \frac{1}{2} &{} \quad \text {when } X_i = Y_i,\\ 1 &{} \quad \text {when } X_i < Y_i \end{array}\right. } \end{aligned}$$

## Supplementary Information

Supplementary Information.

## Data Availability

Citation Data, Code for the BEST analysis and full posterior traces can be found on FairdomHub (https://doi.org/10.15490/FAIRDOMHUB.1.STUDY.1103.2).
