# Supplementary material for: Bayesian estimation reveals that reproducible models in Systems Biology get more citations
Source: Sci Rep. 2023 Feb 15;13:2695. doi: 10.1038/s41598-023-29340-2 (PMC9931699; doi:10.1038/s41598-023-29340-2)
Supplement: Supplementary file 1 — Supplementary Information. [file 41598_2023_29340_MOESM1_ESM.pdf]

Supporting Information: Bayesian estimation reveals that reproducible models in systems biology get more citations

Sebastian Höpfl, Jürgen Pleiss, Nicole E. Radde

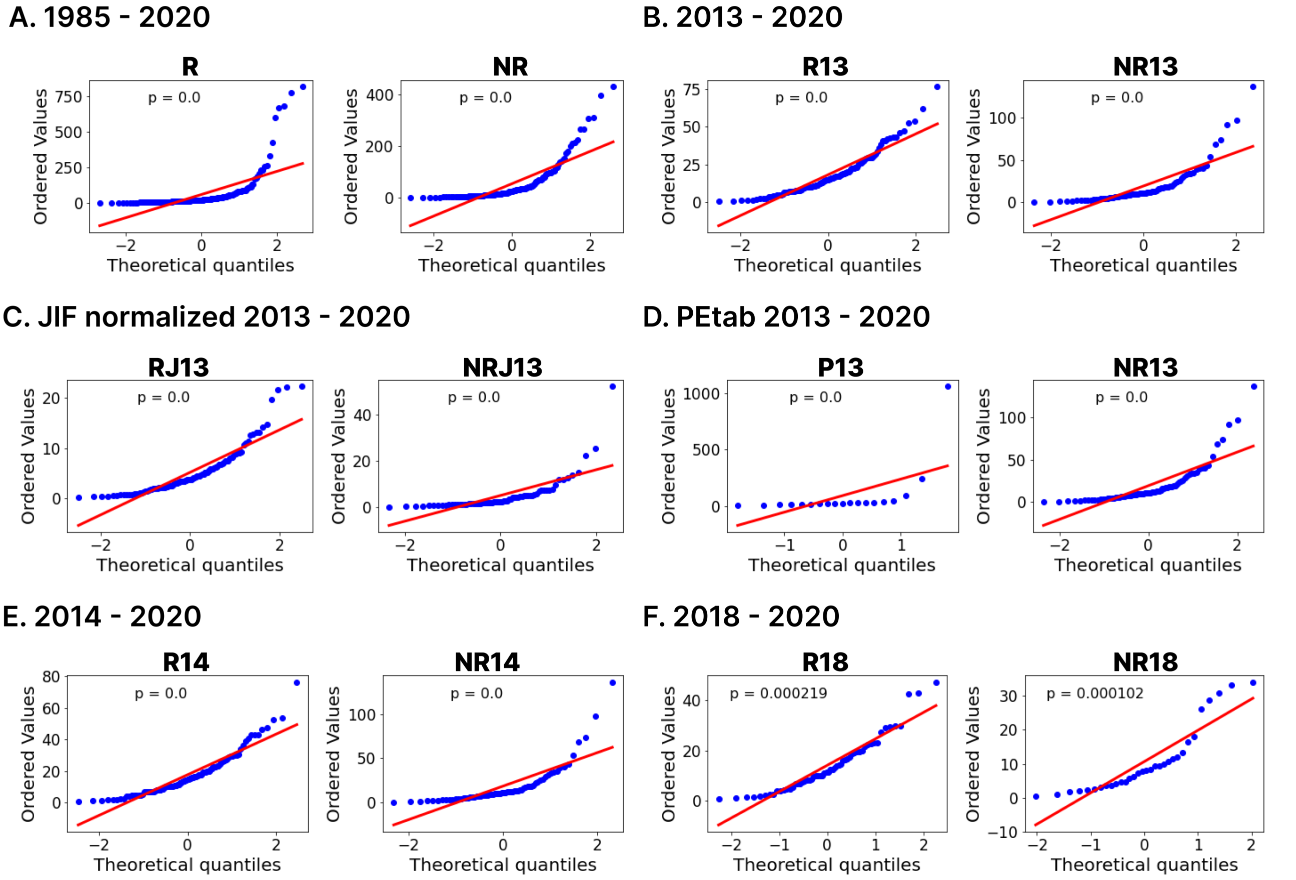

Supplementary Figure 1: The investigated datasets are not normally distributed according to a Shapiro-Wilk test and QQ-plot investigation. QQ-plots of the quantiles of the data against the quantiles of the normal distribution are shown for each comparison with the p-values of the Kolmogorov-Smirnov test for the goodness of the fit to the normal distribution. p-values were rounded to six digits, this led to a value of 0 except for G. A. Citations of papers with reproducible vs. non-reproducible models 1985-2020. B. Citations of papers with reproducible vs. non-reproducible models published between 2013 and 2020. C. JIF normalized citations of papers with reproducible vs. non-reproducible models published between 2013 and 2020. D. Citations of papers of the PEtab benchmark database vs. papers with non-reproducible models between 2013 and 2020. E. Citations of papers with reproducible vs. non-reproducible models published between 2014 and 2020. F. Citations of papers with reproducible vs. non-reproducible models published between 2018 and 2020.

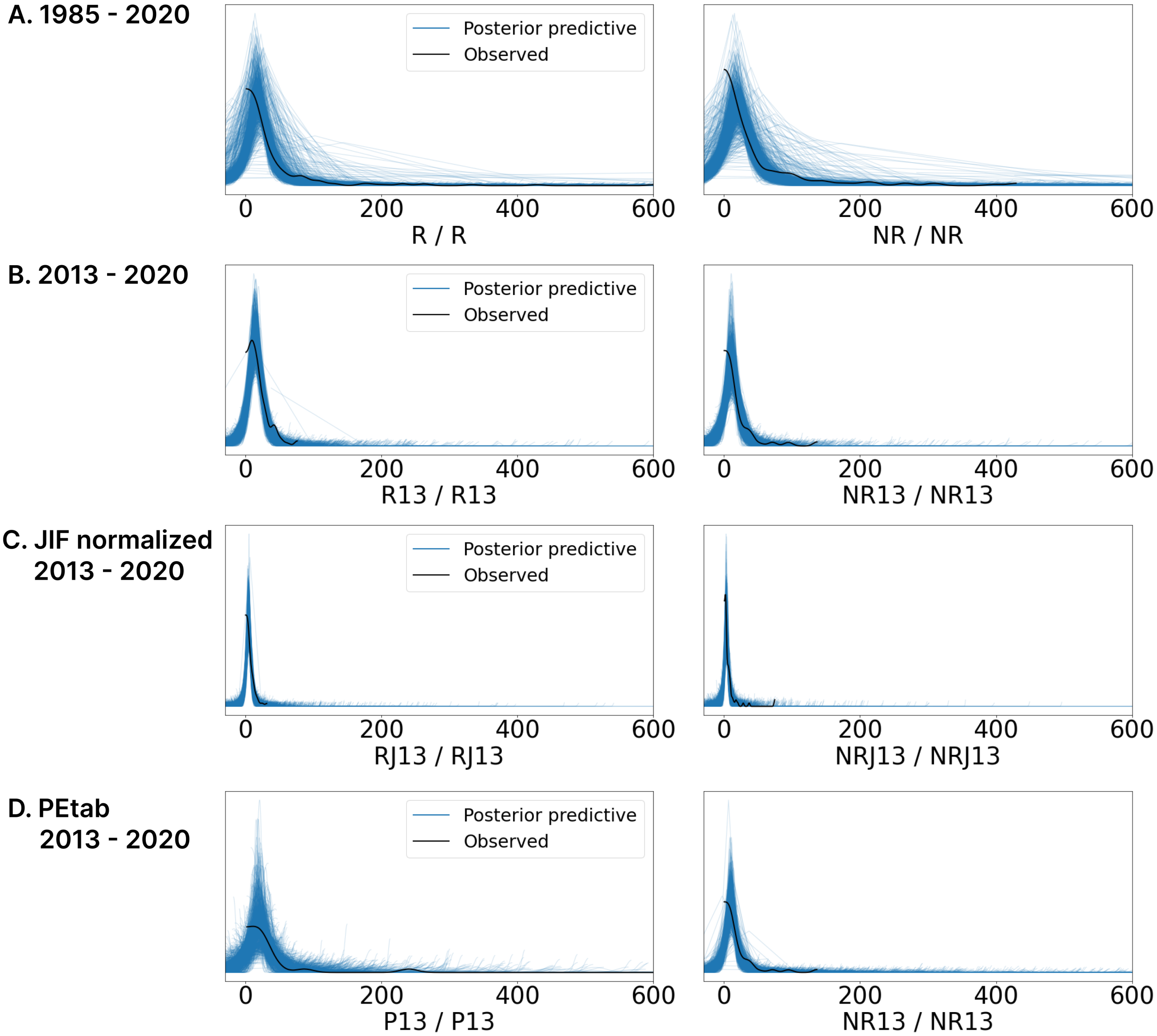

Supplementary Figure 2: Posterior predictive check shows a good agreement of the posterior predictive samples with the observed data. For each dataset, 1000 posterior predictive samples (blue) are shown against the kernel density estimate of the observed data (black). A. Posterior predictive samples of the 1985-2020 dataset. B. Posterior predictive samples of the 2013-2020 dataset. C. Posterior predictive samples of the JIF normalized 2013-2020 dataset. D. Posterior predictive samples of the PEtab and non-reproducible 2013-2020 dataset.

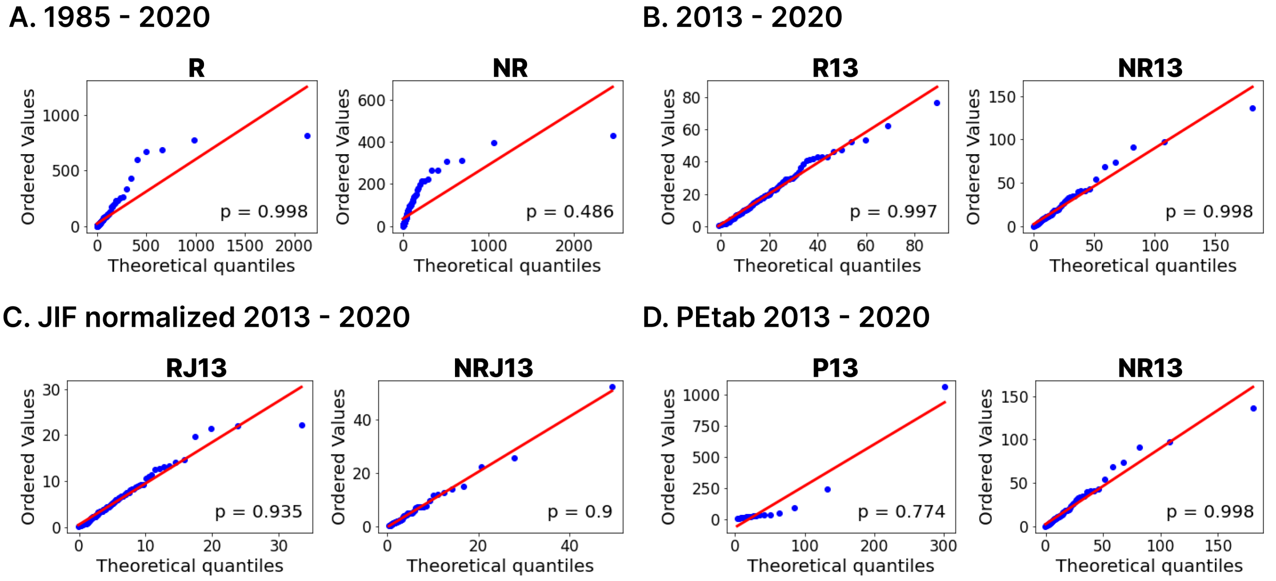

Supplementary Figure 3: All investigated datasets can be described by a non-central t distribution (NCT). QQ-plots of the quantiles of the data against the quantiles of the NCT distribution are shown for each comparison with the p-values of the Kolmogorov-Smirnov test for the goodness of the fit to the NCT distribution. A. Citations of papers with reproducible vs. non-reproducible models 1985-2020. B. Citations of papers with reproducible vs. non-reproducible models published between 2013 and 2020. C. JIF normalized citations of papers with reproducible vs. non-reproducible models between 2013 and 2020. D. Citations of papers of the PEtab benchmark database vs. papers with non-reproducible models between 2013 and 2020.

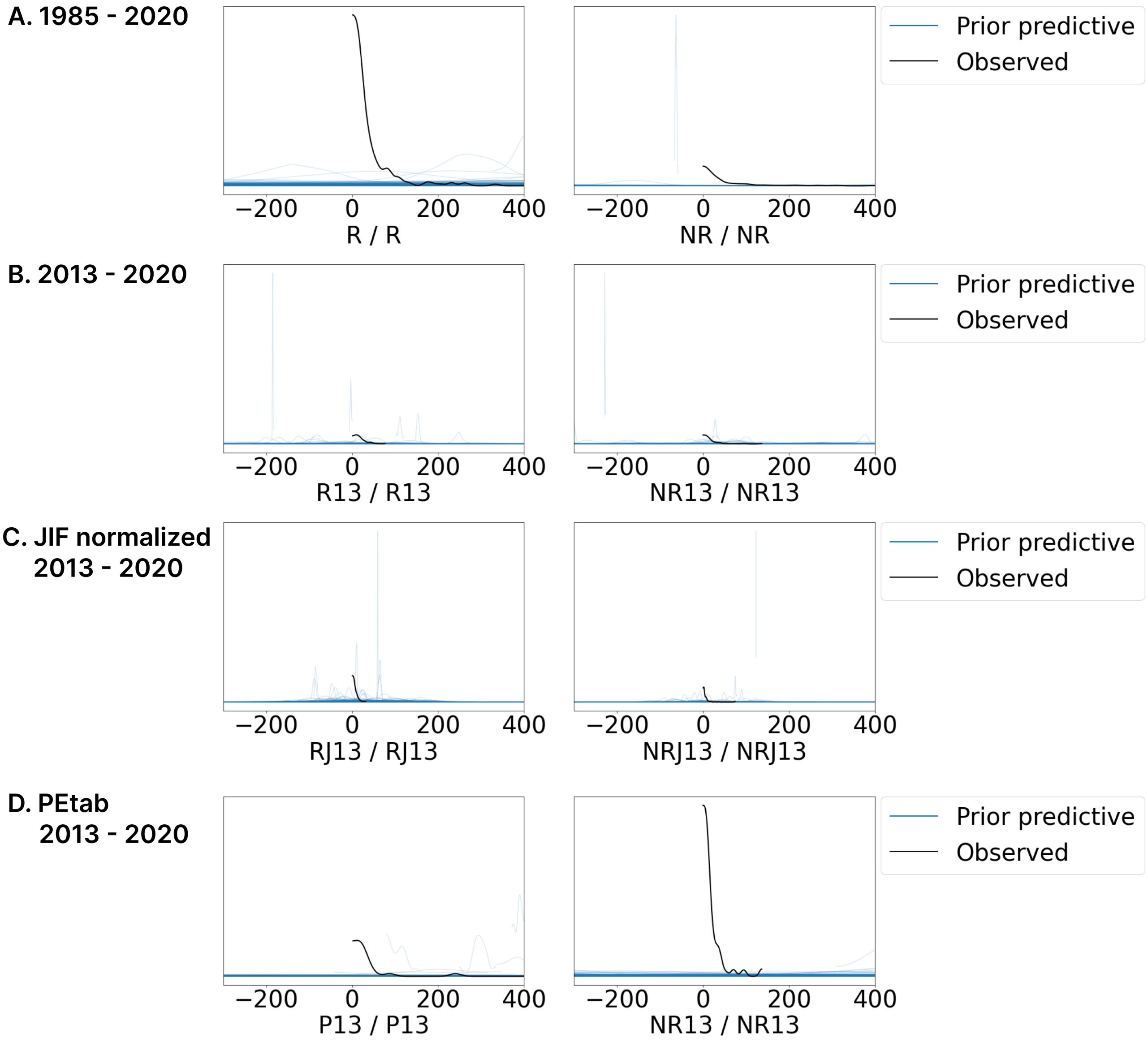

Supplementary Figure 4: The prior can capture the observed data and is uninformed. For each dataset, 1000 prior predictive samples (blue) are shown against the kernel density estimate of the observed data (black). A. Prior predictive samples of the 1985-2020 dataset. B. Prior predictive samples of the 2013-2020 dataset. C. Prior predictive samples of the JIF normalized 2013-2020 dataset. D. Prior predictive samples of the PEtab and non-reproducible 2013-2020 dataset.

Supplementary Table 1: Potential Scale Reduction Factors (PSRF) and Effective Sample Size for all inferred parameters. 100000 were drawn for each chain.

**Dataset**	**Parameters**	**PSRF**	**ESS**
1985-2020 R vs. NR	μ_R	1	3.254e+05
	μ_{NR}	1	2.884e+05
	σ_R	1	2.947e+05
	σ_{NR}	1	2.706e+05
	$\nu_{R,NR}$	1	2.663e+05
	$\mu_R - \mu_{NR}$	1	3.399e+05
	$\sigma_R - \sigma_{NR}$	1	3.947e+05
	$(\mu_R - \mu_{NR})/\sqrt{\frac{(n_R-1)\cdot\sigma_R^2+(n_{NR}-1)\cdot\sigma_{NR}^2}{n_R+n_{NR}-2}}$	1	3.451e+05
2013-2020 R13 vs. NR13	μ_{R13}	1	3.166e+05
	μ_{NR13}	1	2.603e+05
	σ_{R13}	1	2.815e+05
	σ_{NR13}	1	2.282e+05
	$\nu_{R13,NR13}$	1	2.079e+05
	$\mu_{R13} - \mu_{NR13}$	1	3.367e+05
	$\sigma_{R13} - \sigma_{NR13}$	1	3.958e+05
	$(\mu_{R13} - \mu_{NR13})/\sqrt{\frac{(n_{R13}-1)\cdot\sigma_{R13}^2+(n_{NR13}-1)\cdot\sigma_{NR13}^2}{n_{R13}+n_{NR13}-2}}$	1	3.095e+05
2013-2020 RJ13 vs. NRJ13	μ_{RJ13}	1	2.631e+05
	μ_{NRJ13}	1	2.493e+05
	σ_{RJ13}	1	2.352e+05
	σ_{NRJ13}	1	2.329e+05
	$\nu_{RJ13,NRJ13}$	1	2.006e+05
	$\mu_{RJ13} - \mu_{NRJ13}$	1	3.257e+05
	$\sigma_{RJ13} - \sigma_{NRJ13}$	1	4.147e+05
	$(\mu_{RJ13} - \mu_{NRJ13})/\sqrt{\frac{(n_{RJ13}-1)\cdot\sigma_{RJ13}^2+(n_{NRJ13}-1)\cdot\sigma_{NRJ13}^2}{n_{RJ13}+n_{NRJ13}-2}}$	1	3.063e+05
2013-2020 P vs. NR	μ_{P13}	1	3.31e+05
	μ_{NR13}	1	3.382e+05
	σ_{P13}	1	3.539e+05
	σ_{NR13}	1	2.968e+05
	$\nu_{P13,NR13}$	1	2.815e+05
	$\mu_{P13} - \mu_{NR13}$	1	3.314e+05
	$\sigma_{P13} - \sigma_{NR13}$	1	4.178e+05
	$(\mu_{P13} - \mu_{NR13})/\sqrt{\frac{(n_{P13}-1)\cdot\sigma_{P13}^2+(n_{NR13}-1)\cdot\sigma_{NR13}^2}{n_{P13}+n_{NR13}-2}}$	1	3.473e+05
